# A Change in Scope: Endoscopic Retrograde Cholangiopancreatography via a Gastroscope in Billroth II Gastrojejunostomy

**DOI:** 10.7759/cureus.41793

**Published:** 2023-07-12

**Authors:** Lahari Vudayagiri, Hunter L Markle, Daniel Wood, Grant Hubbard, Walter Chlysta

**Affiliations:** 1 Department of Surgery, Western Reserve Hospital, Cuyahoga Falls, USA; 2 Department of General Surgery, Lake Erie College of Osteopathic Medicine, Erie, USA; 3 Department of General Surgery, Lake Erie College of Osteopathic Medicine, Seton Hill, USA

**Keywords:** cbd exploration, forward-viewing endoscope, duodenoscope, gastroscope, choledocholithiasis, endoscopic retrograde cholangio-pancreatography (ercp)

## Abstract

Previous gastric procedures often make endoscopic interventions challenging. Our case study focuses specifically on performing an endoscopic retrograde cholangiopancreatography (ERCP) through a gastroscope (EVIS EXERA III GIF-HQ190, Olympus, Center Valley, USA) in a patient with a history of Billroth II gastrojejunostomy. Successful ERCP in Billroth II using a gastroscope with traditional ERCP instrumentation has been very rarely reported in case reports in the literature review. This case study provides an alternative method of access to the common bile duct (CBD) and treatment of obstruction to prevent the risk of morbidities from an open CBD exploration. The primary diagnosis for this patient was choledocholithiasis. He initially underwent a standard ERCP with a side-viewing duodenoscope (EVIS EXERA II, TJF-Q190V, Olympus, Center Valley, USA); however, due to the difficult anatomy from his previous Billroth II reconstruction, the CBD was very difficult to access. A gastroscope was then used instead to perform the ERCP, providing an innovative endoscopic therapy. Given the patient’s multiple comorbidities, he was at high risk for morbidity and mortality with an open CBD exploration. Hence, this case report provides insight into an innovative endoscopic approach to CBD exploration with difficult anatomy.

## Introduction

Billroth II gastrojejunostomy has long been a known surgical procedure used for gastric ulcer disease and gastric cancers. The procedure itself involves a partial gastrectomy with anastomosis of the gastric antrum to the jejunum. With a Billroth II procedure, there are two limbs created: an afferent and efferent limb. The afferent limb includes the duodenum and receives biliary drainage, the efferent limb continues to the distal small bowel from the area of the jejunum. During standard endoscopic retrograde cholangiopancreatography (ERCP), a duodenoscope is inserted through the mouth and stomach to the second part of the duodenum where the major duodenal ampulla can be visualized. The standard ERCP endoscope is a side-viewing duodenoscope as opposed to a forward-viewing gastroscope. ERCP becomes technically challenging in patients who have undergone Billroth II gastrojejunostomy for several reasons. Common sources of failure include difficulty entering the afferent loop and difficulty passing the scope into the duodenum, iatrogenic bowel perforation, and inability to cannulate the common bile duct (CBD) [1].

To address the difficulty of entering the afferent loop and duodenum, the duodenoscope can be exchanged for a thinner, more flexible gastroscope at the expense of a narrower instrument channel and the loss of an elevator. Some studies have shown increased success with major duodenal ampulla cannulation when using a gastroscope for ERCP in Billroth II patients compared to a duodenoscope [2,3]. However, another study demonstrated a lower rate of successful CBD cannulation when using a gastroscope, yet higher rates of successfully intubating the afferent limb [4]. This demonstrates the advantage of the gastroscope’s flexibility and smaller diameter in navigating the challenging anatomy after Billroth II and reaching the duodenal papilla; however, it may also highlight the more difficult instrument manipulation with the absence of an elevator, suggested by the lower cannulation rate.

Another consideration when using a gastroscope for ERCP is the narrower instrument channel compared to a duodenoscope; instruments commonly used in ERCP and designed traditionally to be used with a duodenoscope may not be readily compatible with a gastroscope. While there are reports on ERCP and sphincterotomy using a gastroscope, there is less literature on other interventions such as endoscopic papillary balloon dilation (EPBD) [5]; no reports of stone extraction using a retrieval basket with a gastroscope could be found in the literature review.

## Case presentation

The patient was an 83-year-old male with a past medical history of dementia, type 2 diabetes mellitus, hypertension, and hyperlipidemia. He was a poor historian with no reliable point of contact for history review. He noted a history of a “stomach procedure” but was unsure of his surgical history. A thorough chart view did not identify any specific surgical history. The patient initially presented to the emergency department with increased confusion and malnutrition.

**Figure 1:**
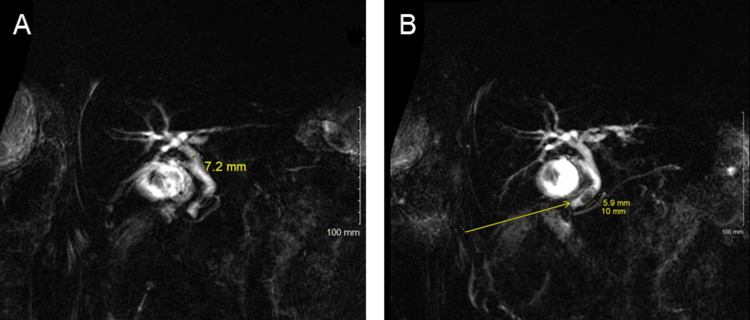
MRCP images (A) CBD diameter of 7 mm. (B) Filling defect in the distal CBD measuring 6 x 10 mm. MRCP: magnetic resonance cholangiopancreatography, CBD: common bile duct

On physical exam, his abdomen was not concerned for any acute pathology; however, his labs were significant for transaminitis of AST/ALT 604/661 U/L and elevated lactic acid to 3.1 mmol/L. A right upper quadrant ultrasound showed cholelithiasis and sludge with a mildly thickened gallbladder wall. The CBD on the ultrasound measured 7mm. He subsequently underwent a magnetic resonance cholangiopancreatography (MRCP), which demonstrated cholelithiasis and choledocholithiasis with a filling defect 1 cm above the distal end of the CBD without biliary dilation (Figure 1A-1B). Of note, a computed tomography (CT) scan of the abdomen and pelvis was not completed secondary to the patient’s benign abdominal exam. The patient underwent an ERCP due to choledocholithiasis with concern for the possible development of cholangitis.

**Figure 2:**
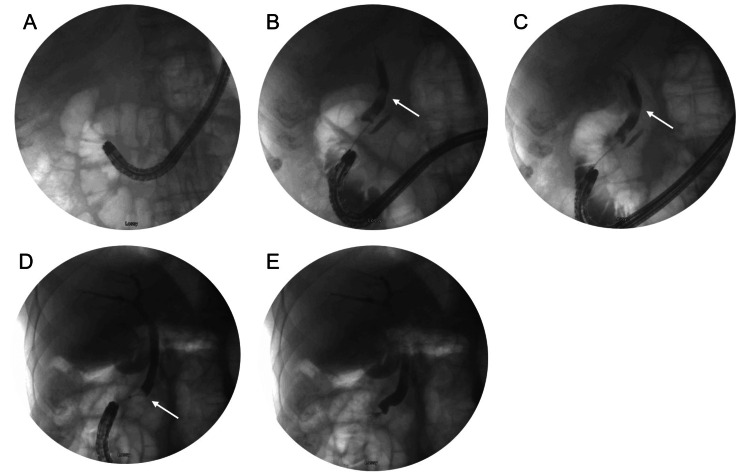
ERCP cholangiogram images (A) Gastroscope intubation of the afferent limb. (B) Guidewire cannulation of the CBD showing 1.0 x 0.5 cm stone. (C) Advancement of the guidewire past the obstruction. (D) Entrapment of the stone within the FlowerBasket. (E) Post-extraction cholangiogram demonstrating no residual CBD obstruction. ERCP: endoscopic retrograde cholangiopancreatography, CBD: common bile duct

**Figure 3:**
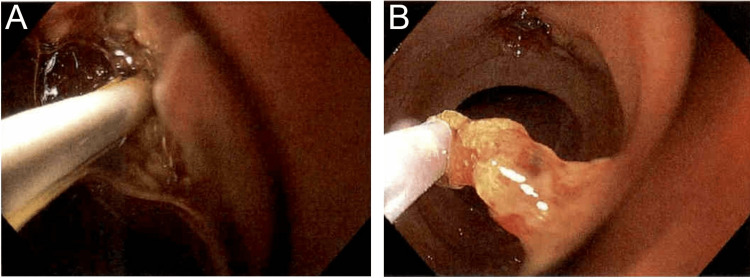
Forward-viewing gastroscope images (A) Sphincterotomy with the Hydratome RX 44. (B) Entrapment of the obstructing stone in the FlowerBasket following extraction from the CBD. CBD: common bile duct

The patient was placed supine and general anesthesia was employed. A duodenoscope (EVIS EXERA II, TJF-Q190V, Olympus, Center Valley, USA) was inserted without difficulty into the stomach, at which point it became apparent the patient had had an antrectomy with Billroth II anastomosis. The efferent and afferent limbs were not easily differentiated. Each limb was explored with the duodenoscope, but due to angulation, the scope could not accurately identify the ampulla of Vater. A gastroscope (EVIS EXERA III GIF-HQ190, Olympus, Center Valley, USA) was then used to identify the afferent limb. There was initial difficulty with advancing the scope into the afferent limb, but eventually, the duodenal stump was reached and the ampulla was identified (Figure 2A). The ampulla was cannulated with a wire and a Hydratome RX 44 sphincterotome (.035"/260cm, Boston Scientific, Marlborough, USA) was advanced into the duct. A cholangiogram showed a 1.0 x 0.5 cm distal stone but otherwise normal ductal anatomy (Figure 2B-2C). A sphincterotomy was performed at approximately the 6 o’clock position accounting for the patient’s supine positioning (Figure 3A). Next, we attempted to advance an Extractor Pro RX Triple-lumen Retrieval Balloon (Boston Scientific, Marlborough, USA) over the guidewire; however, it was too thick for the channel of the gastroscope. The wire from a Jagtome Revolution RX 39 sphincterotome (.025”/260cm, Boston Scientific, Marlborough, USA) was used to cannulate the duct. As this wire had a smaller diameter, the retrieval balloon was able to be threaded over this wire and through the gastroscope channel. Some debris was removed, but the filling defect persisted. The balloon and wire were removed, and a FlowerBasket stone retrieval basket (8 WIRE, 190cm X 20mm, Olympus, Center Valley, USA) was advanced into the duct (Figure 2D). We were able to fragment the stone and witness its removal (Figure 3B). We removed the remaining debris with the balloon and performed a balloon occlusion cholangiogram which showed no filling defect (Figure 2E).

The patient recovered well, and his transaminitis improved. He was discharged to a skilled nursing facility.

## Discussion

ERCP is a challenging procedure to successfully perform in patients with a history of Billroth II gastrectomy. In a retrospective study of 110 patients with a history of Billroth II undergoing a total of 185 ERCP attempts, Faylona et al. report an ERCP failure rate of 34%. Reasons for failure included difficulty entering the afferent loop and difficulty passing the scope into the duodenum, iatrogenic bowel perforation, and inability to cannulate the CBD [1]. In this case, the initial attempt of ERCP was performed unsuccessfully with a duodenoscope due to difficulty identifying the afferent limb and subsequently, the ampulla of Vater. Trying again with a gastroscope, the afferent limb and ampulla of Vater were identified.

Kim et al. compared ERCP and sphincterotomy using a traditional duodenoscope and a gastroscope. In the 22 patients who underwent ERCP with a duodenoscope, the major duodenal papilla was successfully cannulated in 68% of cases compared to an 87% success rate in the 23 cases that utilized a gastroscope. After cannulation, there was little difference in the success of sphincterotomy between the duodenoscope and gastroscope (80% versus 83%) [2]. This study highlights one of the major advantages of the gastroscope in ERCP: its narrower diameter and increased flexibility compared to the duodenoscope allow for easier manipulation through challenging anatomy.

Another challenge presented by the gastroscope is its narrower instrument channel. The Olympus GIF-HQ190 gastroscope has a 2.8 mm instrument channel diameter compared to the 4.2 mm diameter of the Olympus TJF-Q190V duodenoscope. As we described in our report, we were successful in adapting instruments traditionally used with a duodenoscope to work through a gastroscope and remove the CBD obstruction in our patient. Utilizing EPBD through a gastroscope, which was previously reported by Park et al., and a stone retrieval basket, of which no prior report could be found, we demonstrated that advanced ERCP instrumentation can be used with a gastroscope and is a potential new approach to increase ERCP success rate in patients with a history of Billroth II gastrectomy [5].

Worth mentioning, laparoscopic common bile duct exploration (LCBDE) is an effective procedure to remove CBD obstructions in patients when ERCP has failed, especially in Billroth II patients who are known to present challenges with ERCP. In a retrospective study, Choi et al. compared the use of LCBDE in patients with no or unrelated abdominal procedures to patients with a history of gastrectomy. They determined there to be no significant difference between the groups in regard to both perioperative clinical outcomes and postoperative complications. They claim that LCBDE is the best alternative to failed ERCP in complicated choledocholithiasis cases [6]. However, in patients with multiple comorbidities who are poor surgical/general anesthesia candidates, adaptations to increase the success of ERCP may allow LCBDE to be avoided altogether.

Rogers et al. compared outcomes in low-risk patients with cholecystolithiasis and choledocholithiasis who received either laparoscopic cholecystectomy and LCBDE or ERCP sphincterotomy plus laparoscopic cholecystectomy and found the procedures were equally effective in removing the stone. Furthermore, both options showed similar overall cost, patient acceptance, and quality of life scores. They did find that the group who received laparoscopic cholecystectomy and LCBDE had a significantly shorter length of stay and physician stay [7]. Little data exist regarding the morbidity and mortality of either procedure in high-risk patients. In this case, we present an approach for stone clearance by modified ERCP in patients who would be poor surgical candidates and may not tolerate general anesthesia.

## Conclusions

There are several advantages to adapting the ERCP technique by using a gastroscope in patients with challenging anatomy. Compared to other interventions for CBD obstruction, ERCP can be completed without general anesthesia, which may be beneficial in select patient populations. Additionally, ERCP is less invasive than surgical interventions and generally allows for a quicker return to normal activities. In cases like ours where ERCP was in progress prior to learning of the patient's Billroth procedure, adapting by using a gastroscope and continuing with ERCP rather than preparing for and converting to another procedure may save time and allow for a shorter hospital course.

While there are advantages to modifying the ERCP technique, doing so also comes with its own set of challenges. Success is highly dependent on the skill of the physician, and the inherent differences between the gastroscope and duodenoscope (for example the absence of an elevator) may complicate the technique for less experienced physicians. Additionally, operative time may increase when trying to adapt the procedure and the instruments to work through the narrower channel of the gastroscope, leading to increased costs and decreased operating room efficiency. Lastly, the instruments and scopes available vary significantly between hospitals. Consequently, there may be cases when no instruments are on hand that can fit through the gastroscope channel, prohibiting the success of this technique.

ERCP can be challenging to perform in patients with difficult anatomy. This case report demonstrates a novel approach to ERCP in patients with difficult anatomy thus sparing the potential risk of having to undergo LCBDE or an open CBD exploration. The use of balloon and wire cage stone fragmentation guided by a gastroscope rather than a duodenoscope may be a way to increase the success rate of ERCP in patients with Billroth II anatomy.
